# Myzomyia and Pyretophorus series of *Anopheles* mosquitoes acting as probable vectors of the goat malaria parasite *Plasmodium caprae* in Thailand

**DOI:** 10.1038/s41598-022-26833-4

**Published:** 2023-01-04

**Authors:** Anh Hoang Lan Nguyen, Sittiporn Pattaradilokrat, Winai Kaewlamun, Osamu Kaneko, Masahito Asada, Morakot Kaewthamasorn

**Affiliations:** 1 The International Graduate Program of Veterinary Science and Technology (VST), Faculty of Veterinary Science, Chulalongkorn University, Bangkok, 10330 Thailand; 2 Veterinary Parasitology Research Unit, Faculty of Veterinary Science, Chulalongkorn University, Bangkok, 10330 Thailand; 3 Department of Biology, Faculty of Science, Chulalongkorn University, Bangkok, 10330 Thailand; 4 School of Agricultural Resources, Chulalongkorn University, Bangkok, Thailand; 5 Department of Protozoology, Institute of Tropical Medicine (NEKKEN), Nagasaki University, Nagasaki, 852-8523 Japan; 6 National Research Center for Protozoan Diseases, Department of Global Cooperation, Research Unit for Global Infection Control, Obihiro University of Agriculture and Veterinary Medicine, Obihiro, 080-8555 Japan

**Keywords:** Ecology, Microbiology, Molecular biology, Diseases

## Abstract

Unlike malaria parasites in humans, non-human primates, rodents, and birds, ungulate malaria parasites and their vectors have received little attention. As a result, understanding of the hosts, vectors, and biology of ungulate malaria parasites has remained limited. In this study, we aimed to identify the vectors of the goat malaria parasite *Plasmodium caprae*. A total of 1019 anopheline and 133 non-anopheline mosquitoes were collected from goat farms in Thailand, where *P. caprae*-infected goats were discovered. Anopheline mosquitoes were identified using molecular biological methods that target the *cytochrome c oxidase subunit 1* (*cox1*), the *cytochrome c oxidase subunit 2* (*cox2*) genes, and the internal transcribed spacer 2 (ITS2) region. Pool and individual mosquitoes were tested for *P. caprae* using the head-thorax parts that contain the salivary glands, with primers targeting three genetic markers including *cytochrome b*, *cytochrome c oxidase subunit 1*, and *18S small subunit ribosomal RNA* genes. Additionally, goat blood samples were collected concurrently with mosquito surveys and screened to determine the status of malaria infection. This study revealed nine mosquito species belonging to six groups on goat farms, including Hyrcanus, Barbirostris, Subpictus, Funestus, Tessellatus, and Annularis. The DNA of *P. caprae* was detected in *Anopheles subpictus* and *Anopheles aconitus.* This is the first time *An. subpictus* and *An. aconitus* have been implicated as probable vectors of *P. caprae*.

## Introduction

Humans and various animals are susceptible to malaria, a mosquito-borne disease, so it has been investigated for centuries. The parasites responsible have been researched and understood thoroughly, and the disease has been well-managed to ensure public health. On the other hand, ungulate malaria parasites have undergone a few research studies, primarily based on molecular approaches^1–5^. Among *Plasmodium* spp. infecting even-toed ungulates (Order Artiodactyla), *Plasmodium cephalophi* and *P. brucei* were first described in duiker antelope in Africa (1913)^6^. Later, *P. bubalis* was found in water buffalo (Bovidae: *Bubalus bubalis*) in India (1919)^7^ and other Asian countries, including Vietnam (2010 & 2013), Thailand (2014 & 2015), and Nepal (2017)^4,8^. Several years after the initial detection of *P. bubalis, P. caprae* was reported in wild goats (Bovidae: *Capra aegagrus hircus*) in Angola (1923)^9^, it expanded to domestic goats (Bovidae: *Capra aegagrus hircus*) in Zambia (2010), Sudan (2014), Thailand (2016), Myanmar (2016), Iran (2017) and Kenya (2017)^4,10^. Furthermore, *P. traguli* was detected in mouse deer (Tragulidae: *Tragulus javanicus*) in Malaysia (1962)^11^ and *P. odocoilei* in American white-tailed deer (Cervidae: *Odocoileus virginianus*) (1967)^12,13^. Among them, at least three species of malaria parasites are endemic in Southeast Asia, suggesting the presence of mosquito vectors in this region. Although most of these parasites were discovered a long time ago, the descriptions of mosquito vectors have been limited and relied solely on morphology. Thus, a comprehensive picture of malaria parasites and their mosquito vectors remains incomplete.

Thailand is well known as one of the most biodiversity-rich tropical countries in the world. According to Rattanarithikul et al. (2006) and the Walter Reed Biosystematics Unit data repository^14,15^, there are at least 464 mosquito species, with 83 belonging to the genus *Anopheles*. Most mosquito vectors for malaria transmission in Southeast Asia belong to cryptic species complexes that are nearly impossible to microscopically identify owing to their overlapping morphological characteristics^16^. For example, the Barbirostris Complex has six species, five of which exist in Thailand^17–19^. In contrast, the Minimus complex consists of two sibling species (A and C) and has been incriminated as human malaria vectors in Thailand and Southeast Asia^20^. Mosquito determination of species complexes based on morphological characteristics is likely to misidentify and mislead vector incrimination as a result^21,22^.

Despite the limitations and difficulties involved in the identification of mosquito vectors, several previous studies have reported on *Plasmodium* spp. in ungulates. Wild-caught *Anopheles umbrosus* and *An. letifer* in swamp forests were found to carry the *Plasmodium* sporozoites and oocysts in their salivary glands and midguts. These sporozoites and oocysts resembled those of *P. traguli*. Therefore, these mosquitoes were incriminated as probable vectors of *P. traguli* in Malaysian mouse deer^23^. Several decades later, *Plasmodium* spp. lineage B were observed in the salivary glands of sylvatic mosquitoes *An. gabonensis* and *An. obscurus* in the Gabonese forest, Central Africa^2^. *Cytb* sequences from these sporozoites were clustered within the same clade with *Plasmodium* spp. in the African blue duiker and bay duiker^2^. Furthermore, parasites resembling the morphology of *P. odocoilei* were isolated from the salivary glands of *An. punctipennis*. Phylogenetic analysis showed that the DNA sequences of the parasites found in *An. punctipennis* were grouped with *Plasmodium* from white-tailed deer in North America^13^. Recently, a study of malaria in Murrah dairy buffalo revealed that *An. wejchoochotei* or *An. campestris,* and *An. peditaeniatus* were recognised as potential vectors of *P. bubalis* type I in Thailand^24^. However, little is known about the vectors of *P. caprae*, which is also endemic in many other Asian countries. Although several research studies have been carried out on the prevalence, diagnosis, and evolutionary history of *P. caprae* thus far, no information about its vectors has been reported^10,25^. In the present study, we hypothesize that the mosquito vectors of *P. caprae* are endemic in Thailand and other Southeast Asian countries. Hence, this study aimed to identify anopheline mosquitoes as possible vectors of *P. caprae* transmission in Thailand.

## Results

### Detection of *P. caprae* in goat blood samples

A total of 423 goat blood samples were collected from 18 goat farms in three provinces of Thailand at different times, including Kanchanaburi in 2020 (three farms), 2021 (three farms), and 2022 (two farms), Nan in 2020 (five farms) and 2021 (three farms), and Phetchaburi in 2021 (two farms); among which 401 samples were collected simultaneously with mosquito samples from the same farm. Furthermore, 22 blood samples collected in Ratchaburi in 2018 were included in this study. Nested PCR amplification targeting three gene loci, *cytb, cox1,* and *18S rRNA,* identified six *P. caprae* positive samples of 423 blood samples; 2 samples were from Ratchaburi in 2018, 3 samples were from Nan in 2020, and 1 sample was from Phetchaburi in 2021. The PCR products of two *P. caprae* positive samples collected in Ratchaburi in 2018 were further confirmed by Sanger DNA sequencing. BLASTN searches of *cytb* sequences showed 100% identity with *P. caprae* (accession nos. LC326032 & LC090215), 97%–98.0% identity with *P. odocoilei* (accession nos. MH177860 & MK502145), and 96.2% identity with *P. bubalis* (accession nos. LC090213 & LC090214) in the GenBank database. Furthermore, the *cox1* sequences showed 99.6% identity with *P. caprae* (accession nos. LC326032 & LC090215), 98.8% identity with *P. odocoilei* (accession no. OL999536), and 98.0% with *P. bubalis* (accession no. LC090214). The *18S rRNA* sequences revealed a similarity of 92.6% with *P. bubalis* (accession nos. OL624705–OL624709) and 92.2% similarity with *P. falciparum* (accession no. LR131366)*.* The *18S rRNA* sequences from other ungulate malaria parasites are not available in the database. This result confirmed the endemicity of goat malaria in Thailand.

### Species composition of mosquitoes collected from goat farms by morphology

**Figure 1:**
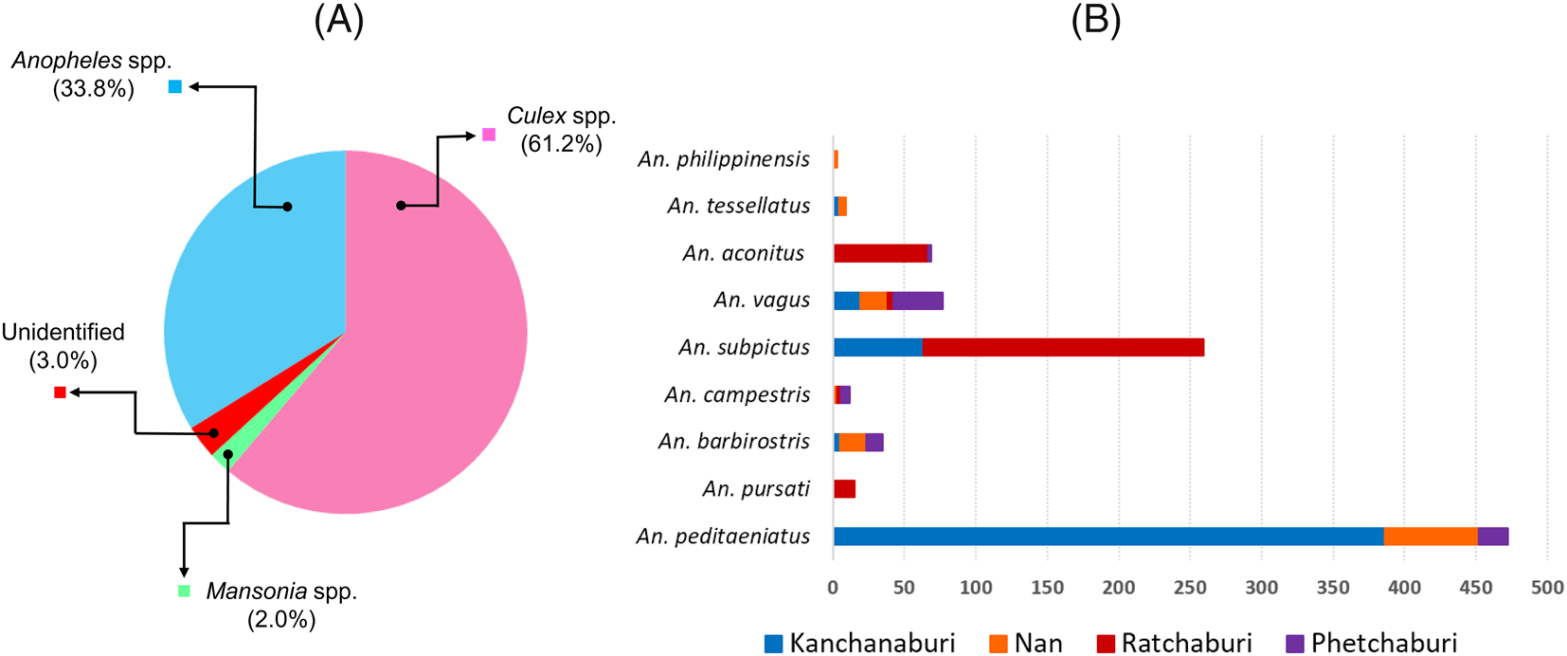
Charts illustrating the composition of mosquitoes determined by morphology. (**A**) The percentages of each genus of mosquitoes collected by CDC light traps in this study. (**B**) The number of mosquitoes of *Anopheles* spp. collected by mouth aspirators in four provinces, including Kanchanaburi, Nan, Ratchaburi and Phetchaburi, as indicated in light green, blue, yellow and dark green, respectively.

Using CDC light traps, a total of 201 female mosquitoes were collected from four goat farms in Kanchanaburi and Phetchaburi, while 951 female anopheline mosquitoes were manually collected by mouth aspirators from eight goat farms in Kanchanaburi, Nan, Ratchaburi, and Phetchaburi provinces during 2020–2021. Morphological examinations of the mosquitoes collected by CDC light traps revealed that 123 (61.2%) were *Culex* spp., 68 (33.8%) were *Anopheles* spp., and 4 (2.0%) were *Mansonia* spp. Six mosquito species (3.0%) remained unidentified due to damage to their wings and legs (Fig. 1A). Among 951 anopheline mosquitoes collected by mouth aspirators, nine mosquito species belonging to six groups/subgroups were identified consisting of *Anopheles peditaeniatus* and *An. pursati* (Hyrcanus), *An. barbirostris* and *An. campestris* (Barbirostris), *An. subpictus* and *An. vagus* (Subpictus), *An. aconitus* (Funestus), *An. tessellatus* (Tessellatus) and *An. philippinensis* (Annularis). Details concerning the species and the number of mosquitoes in each province are illustrated in Fig. 1B.

### Identification of *P. caprae* in anopheline and non-anopheline mosquitoes

Three hundred and twenty-two anopheline mosquitoes were dissected to separate their head and thorax parts containing the salivary glands from the abdomen parts containing the midguts. The salivary glands and midguts were then stained with 0.1% mercurochrome dye, and the presence of oocysts or sporozoites was examined under a microscope. However, no parasites were found. Subsequently, the head and thorax parts containing salivary glands were pooled from one to three samples, depending on the groups and species, to generate 358 pools for DNA extraction. The number of each pool was as follows: Hyrcanus (n = 508, 175 pools), Barbirostris (n = 48, 19 pools), Subpictus (n = 379, 130 pools), Aconitus (n = 72, 27 pools) Tessellatus (n = 9, 5 pools), and Annularis (n = 3, 2 pools). PCR screening targeting *Plasmodium cytb*, *cox1*, and *18S rRNA* genes and following sequencing analysis revealed three pools (0.84%) out of 358 pools were positive for *P. caprae*. These mosquito samples were the Aconitus group (ID THMosGoat21-02_P11) and the Subpictus group (IDs THMosGoat21-01_P18 and THMosGoat21-01_P38) (Table 1). It should be noted that three mosquito pools that were PCR positive to *P. caprae* consisted of two pools of unfed mosquitoes (one pool each of *An. subpictus* and *An. aconitus*) and one pool of blood-fed mosquitoes (*An. subpictus*). Minimum infection rates (MIR) were 1.4% (95% CI 0.25–7.46) in the Aconitus group mosquito and 0.9% (0.25–3.24) in the Subpictus group mosquito (Table 2).

**Table 1:** Summary of the screening of *P. caprae* in anopheline mosquitoes collected from goat farms.

Group	No. collected	No. of pools	No. of positive pools			No. of pools sequenced for determination of mosquito species
			*cytb*	*cox1*	*18S rRNA*	
Hyrcanus	508	175	0	0	0	37 (*An. peditaeniatus*), 3 (*An. pursati*)
Barbirostris	48	19	0	0	0	10 (*An. barbirostris*), 5 (*An. campestris*)
Subpictus	379	130	2	2	2	32 (*An. subpictus*), 20 (*An. vagus*)
Aconitus	72	27	1	1	1	12 (*An. aconitus*)
Tessellatus	9	5	0	0	0	5 (*An. tessellatus*)
Annularis	3	2	0	0	0	2 (*An. philippinensis*)
Total	1,019	358	3	3	3	126

A total of 3 pools of anopheline mosquitoes were positive using PCR assays targeting the *cytb*, *cox1* and *18S rRNA* genes. The number of pools used for species identification was also indicated.

**Table 2:** Minimum infection rates (MIR) of *P. caprae* among collected mosquitoes.

Species	Total no. of mosquitoes	Pool size (range)	No. of tested	No. of positive pools	MIR (%) (95% CI)
*An. aconitus*	72	1–3	72	1	1.4 (0.25–7.46)
*An. subpictus*	296	1–3	221	2	0.9 (0.25–3.24)

From 133 non-anopheline mosquito samples, 8 pools of 25 *Culex* spp. samples and 3 pools from 4 *Mansonia* spp. samples were subjected to PCR examination, as described above. However, none were positive for *Plasmodium* spp.

### Phylogenetic analyses of *P. caprae* in anopheline mosquitoes

**Figure 2:**
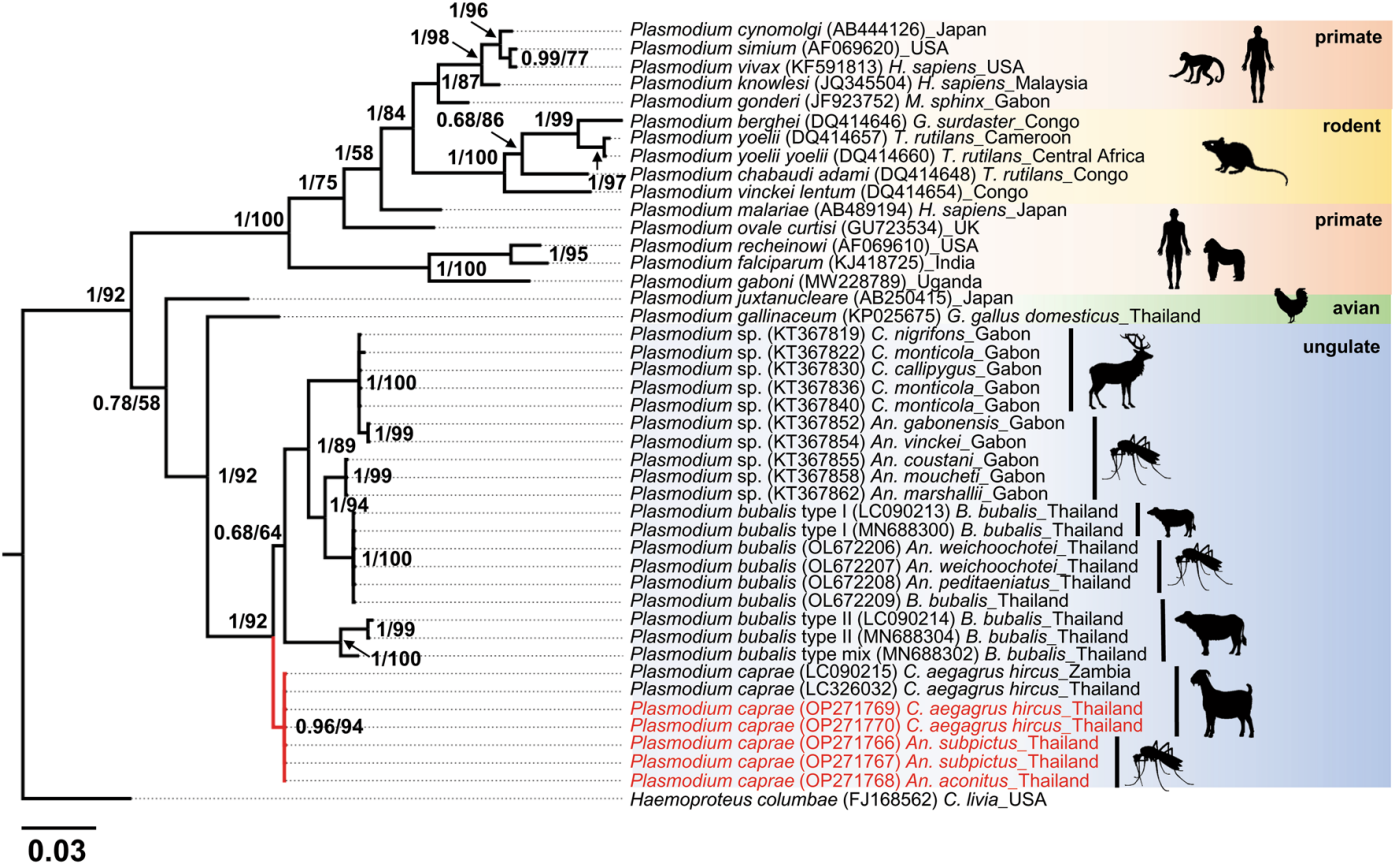
The phylogenetic position of *P. caprae* detected from *Anopheles* mosquitoes and goat blood in this study. The phylogenetic tree was inferred by Bayesian inference using partial *cytb* sequences (632 bp) with the *Haemoproteus columbae* sequence as a root. The posterior probabilities are given by Bayesian inference, and the bootstrap values by Maximum likelihood (≥ 0.68/58) are given in the nodes. Sequences obtained in this study are highlighted in red, and reference sequences retrieved from the GenBank database are highlighted in black. The length of substitutions/site (0.03) is indicated.

BLASTN similarity searches using *cytb* sequences obtained from two pools of *An. subpictus* mosquitoes (THMosGoat21-01_P18 & THMosGoat21-01_P38) and one pool of *An. aconitus* (THMosGoat21-02_P11) revealed 100% similarity to *P. caprae* (accession nos. LC090215 & LC326032) isolated from goats in Zambia and Thailand, 97–98% similarity to *P. odocoilei* (accession nos. MH177860 & LC326035), and 96.2% similarity to *P. bubalis* (accession nos. LC090213 & LC090214). The phylogenetic tree inferred from the *cytb* sequences showed that *Plasmodium* sequences derived from *An. subpictus* (group Subpictus) and *An. aconitus* (Funestus group) were clustered together with *P. caprae* from Thailand and Zambia (accession nos. LC326032 & LC090215) with high Bayesian posterior probability (BPP = 0.96) and bootstrap value (BV = 94) (Fig. 2).

**Figure 3:**
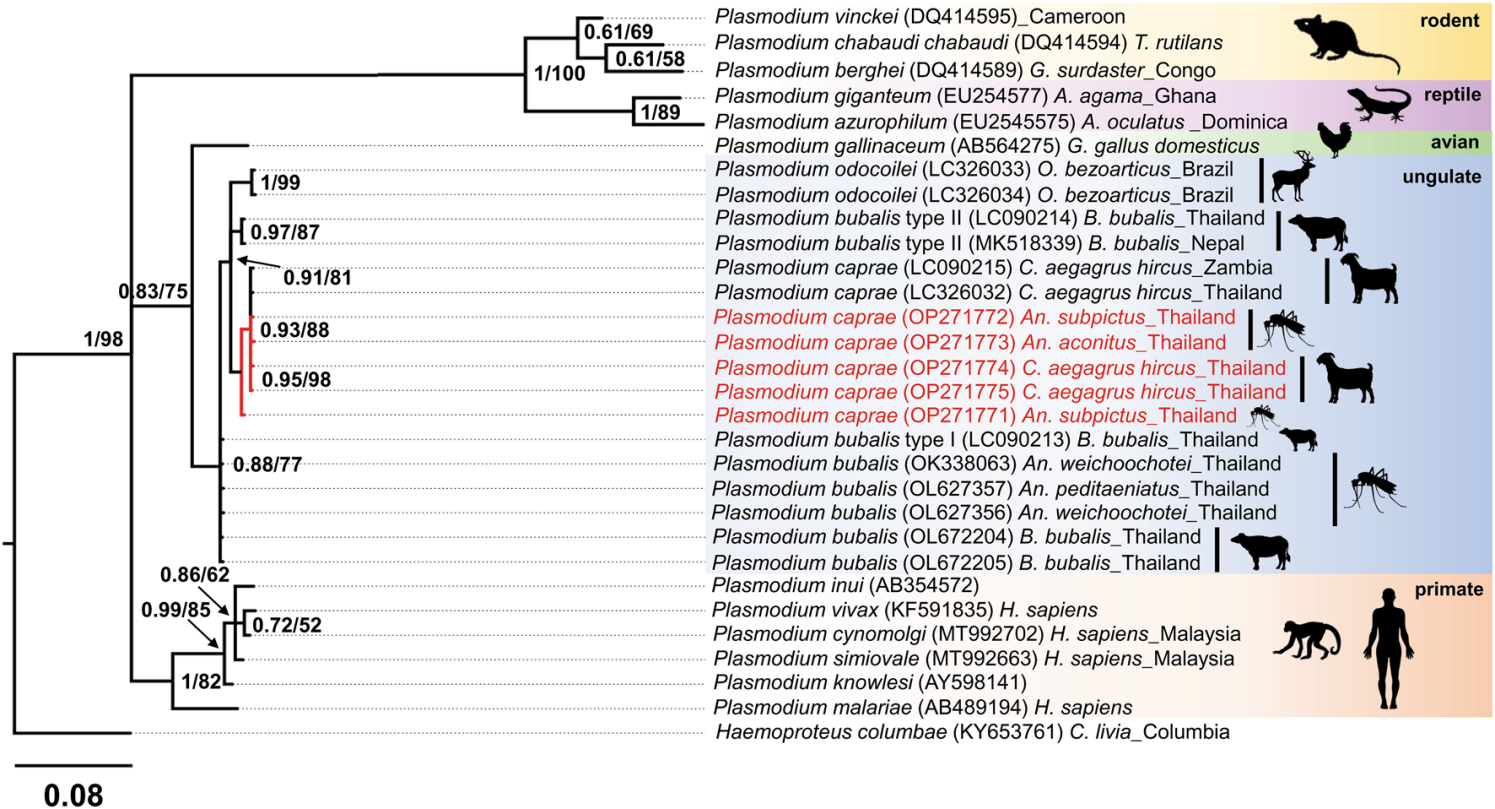
The phylogenetic position of *P. caprae* obtained in this study inferred by Bayesian inference using a partial *cox1* gene (231 bp). All sequences were rooted with *Haemoproteus columbae*. Bayesian posterior probabilities (BPP ≥ 0.61) and bootstrap values (BV ≥ 52) are given at the nodes. The length of the substitutions/site (0.08) is shown.

BLASTN searches of the partial *Plasmodium cox1* sequences of *An. subpictus* and *An. aconitus* showed 100% similarity to the *P. caprae* sequences (accession nos. LC326032 & LC090215), 98–98.8% similarity to *P. odocoilei* (accession nos. LC326034 & OL999536), and 98.2–98.7% similarity to *P. bubalis* (accession nos. LC090214 & LC090213). Similar to the phylogenetic tree inferred from the *cytb* sequences, the *Plasmodium cox1* sequences detected from *An. subpictus* and *An. aconitus* mosquitoes were clustered together with the *P. caprae cox1* sequences from goats in Zambia and Thailand with a BPP value of 0.95 and a BV of 98 (Fig. 3).

**Figure 4:**
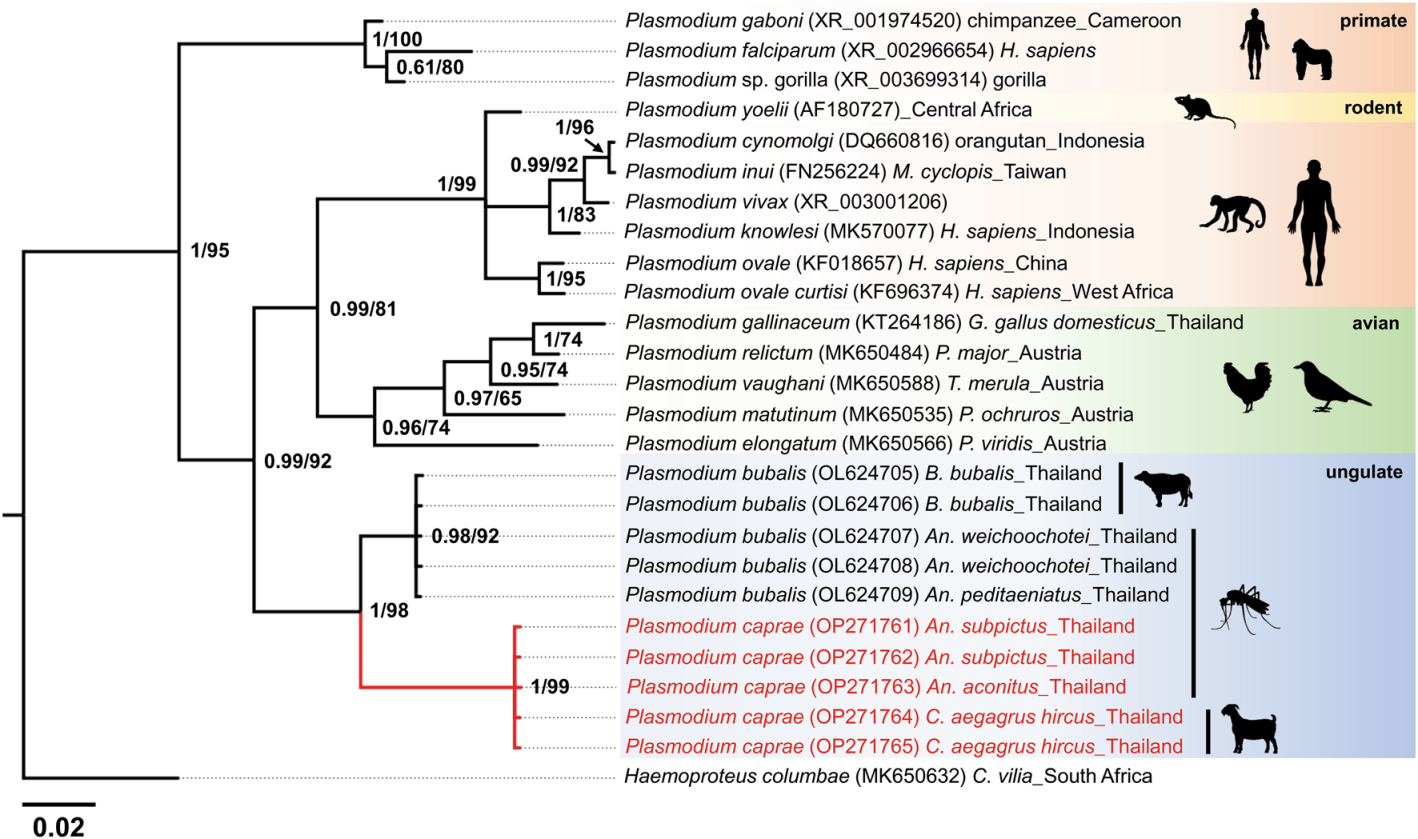
The phylogenetic position of *P. caprae* obtained in this study using a partial *18S rRNA* gene (335 bp). All sequences were rooted with *Haemoproteus columbae*. Posterior probabilities and bootstrap values (≥ 0.61/65) are given in the nodes. The length of the substitutions/site (0.02) is shown.

The *Plasmodium 18S rRNA* sequences obtained from three mosquito pools were identical to the sequences obtained from goats. The phylogenetic tree revealed that all five sequences for *18S rRNA* in this study were clustered in the same clade with *P. bubalis* sequences from water buffaloes and mosquitoes with high support for posterior probabilities of 1 and a bootstrap value of 99 (Fig. 4).

### Molecular identification of anopheline mosquitoes

To identify anopheline mosquito species collected from goat farms using a molecular approach, the partial sequences of *cox1, cox2,* and ITS2 were employed for DNA amplification and sequencing in three *Plasmodium*-positive mosquito pools and 123 negative pools in this study. The sequences obtained were evaluated for BLASTN searches against the GenBank and BOLD databases. A similarity of ≥ 97% for each sequence deposited in the GenBank or BOLD databases was considered the same species. The results were generally consistent among three genetic markers, except for some *cox1* sequences from five species. The results of the *cox1* BLASTN search showed the closely related mosquito species in the same group, while the *cox2* and ITS2 searches presented the same species among three markers.

Regarding *An. aconitus*, the BLASTN search results were in complete agreement among *cox1, cox2* and ITS2. The per cent identity of the *An. aconitus* in this study with reference sequences in GenBank ranged from 97.0 to 99.4% with HQ877378 (*cox1* sequences) and 99.5–100% with JX070686 (*cox2* sequences), while *An. aconitus*-ITS2 sequences showed 100% with MF535233. However, the BLASTN searches for *An. subpictus* had discordant results between *cox2,* ITS2 and *cox1*. In detail, the *An. subpictus*-*cox2* sequences showed 93.6–96.4% similarity to *An. subpictus* in India (KX669656), while the ITS2 sequences showed 99.2%–100% identity to *An. subpictus* in Vietnam (GQ870330). In contrast, *cox1* sequences revealed different species, ranging from 91.9 to 93.3% similarity to *An. epiroticus* (KT382821). However, previous studies on evolutionary divergence suggested that the threshold for intraspecific variation should be 2–3%^26–28^. Therefore, the final identification for this mosquito species was *An. subpictus*, supported by the higher % similarity of the *cox2* and ITS2 sequences.

## Discussion

This study aimed to identify the possible vectors of *P. caprae* in Thailand. We collected mosquitoes from goat farms in four provinces comprising Kanchanaburi, Nan, Ratchaburi, and Phetchaburi in 2020 and 2021. Unfortunately, goat blood collection in Ratchaburi province was not permitted on this trip in June 2021, where we found *P. caprae*-positive mosquitoes. Therefore, 22 goat blood samples previously collected from the same farm in 2018 were rescreened and the sequences of three marker genes were obtained. Five mosquito species were present on the goat farms where *P. caprae*-positive goats were detected, including *An. subpictus, An. aconitus, An. pursati, An. vagus,* and *An. campestris*. These mosquitoes are prevalent not only in Thailand but throughout Asian countries^14^; *An. subpictus* and *An. vagus* exist in Thailand, Vietnam, Cambodia, Indonesia, Malaysia, India and Sri Lanka, whereas *An. aconitus and An. campestris* can be found in Thailand, Vietnam, and Cambodia^29,30^. Among them, *An. subpictus*, *An. vagus,* and *An. aconitus* were recently incriminated for transmitting human malaria parasites^31^.

This study found the DNA of *P. caprae* in two dominant mosquito species of *An. subpictus* and *An. aconitus* of Ratchaburi in 2021. Therefore, these mosquito species are probable vectors of goat malaria parasites. However, no sporozoites were observed in the salivary glands of these mosquitoes. This is most likely due to the low prevalence (2–5%) and very low parasitemia of *P. caprae* in goats^4,10,25^, resulting in a low infection rate and parasite burden in mosquitoes. The results of this study agreed with the findings of previous studies that showed anopheline mosquitoes could be vectors of ungulate malaria parasites. For example, the sporozoites and oocysts of the mouse deer malaria parasite *P. traguli* were found in the salivary glands of wild-caught *An. umbrosus* and *An. letifer*^23^. Additionally, *An. umbrosus* was also found in malaria high-risk areas of Ranong province in southern Thailand^32^. However, none of these species was found in the present study. In another study, the sporozoites of the white-tailed deer malaria parasite *P. odocoilei* were isolated from *An. punctipennis* in North America^13^. Elsewhere, haemosporidian parasites of antelopes and other vertebrates (family Plasmodiidae) were observed in sylvatic anopheline mosquitoes in Gabon, Central Africa^2^. The most recent study reported on the potential vectors of *An. wejchoochotei* or *An. campestris* and *An. peditaeniatus* for the transmission of *P. bubalis* in water buffalo^24^. Therefore, our results provide supporting information that anopheline mosquitoes could be vectors for ungulate malaria transmission in general. However, more concrete evidence is required to conclude the vectors of *P. caprae*. According to Makanga et al., microscopic observation of *Plasmodium* sporozoites in salivary glands and oocysts in the midgut are prerequisites for malaria vector conclusion^33^. Thus, it is suggested that infected mosquitoes be allowed to feed on naive vertebrate animals and observe the blood-stage parasites^23^.

Phylogenetic analysis based on Bayesian inference and Maximum likelihood confirmed that the *Plasmodium* parasites detected from *An. subpictus* and *An. aconitus* in this study were genetically very close and were clustered within the same clade with *P. caprae* previously isolated from goat blood in Thailand and Zambia^4,10^, suggesting that these mosquito species are highly likely vectors for *P. caprae*.

## Conclusion

In this study, *An. subpictus* and *An. aconitus* collected from goat farms were PCR-positive for *P. caprae*. Although it could not be concluded that two mosquito species were natural vectors for goat malaria due to the lack of morphological observation of sporozoites in the salivary glands or oocysts in the midgut, the findings of this study suggest a potential role of *An. subpictus* and *An. aconitus* mosquitoes in the transmission of *P. caprae.*

## Methods

### Sampling site description and mosquito collection

**Figure 5:**
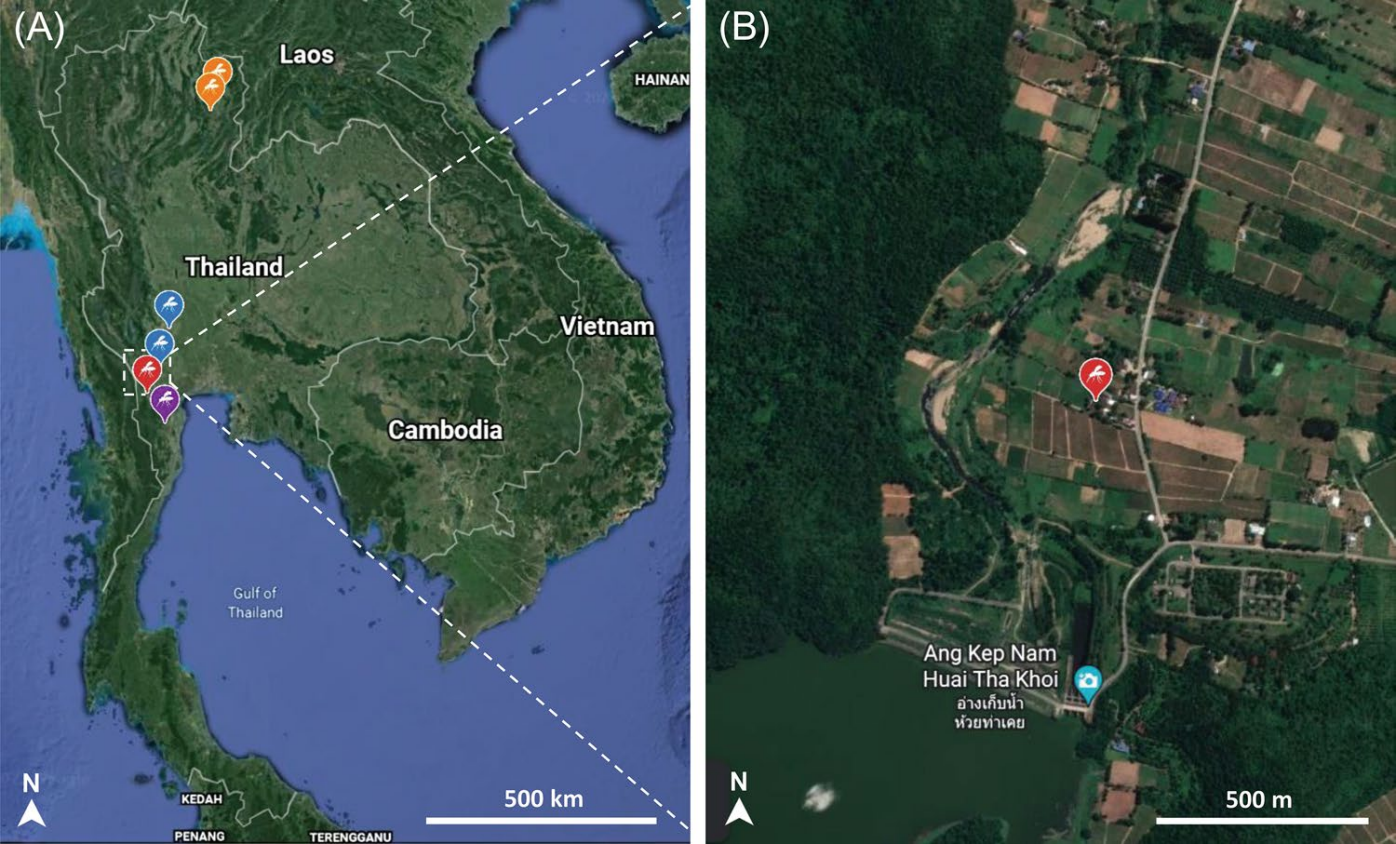
(**A**) Map illustrating the sampling sites in four provinces of Thailand. The orange, blue, red, and purple mosquito silhouettes represent sampling sites in Nan, Kanchanaburi, Ratchaburi, and Phetchaburi provinces. (**B**) A magnified satellite view of mosquito and blood sampling sites in Ratchaburi, where *P. caprae*-positive blood and mosquitoes were found. The images were obtained and modified from Google Earth Pro version 7.3.4.8248 (https://google-earth-pro.updatestar.com/en).

Anopheline and non-anopheline mosquitoes were collected from goat farms in six districts across four provinces of northern and western Thailand during the rainy seasons from June 2020 to November 2021. One or two areas were chosen from each district, including Lao Khwan District (14°28′33.4ʺN 99°48′25.0ʺE and 14°28′51.1ʺN 99°48′11.7ʺE) in Kanchanaburi (2 nights in June 2020), Wiang Sa District (18°31′56.9ʺN 100°37′50.2ʺE) and Mueang Nan District (18°49′07.0ʺN 100°46′36.9ʺE) in Nan (2 nights in August 2020), Ban Kha District (13°17′32.3ʺN 99°25′06.8ʺE) in Ratchaburi (2 nights in June 2021), Kaeng Krachan District (12°53′45.7ʺN 99°42′45.4ʺE) in Phetchaburi (1 night in October 2021), and Ban Mai District in Kanchanaburi (13°53′27.3ʺN99°37′16.2ʺE and 13°54′40.8ʺN 99°37′52.4ʺE) (1 night in November 2021) (Fig. 5A). It should be noted that goat malaria parasites were previously found in Nan, Kanchanaburi, and Phetchaburi^10,25^. Nan is in a high mountainous area that is traversed by the Nan River, while the other three Western provinces are surrounded by dense rainforests (Fig. 5B).

The mosquitoes were collected using CDC light traps and mouth aspirators. For the first method, CDC light traps (John W. Hock Co., Gainesville, FL, USA) were placed approximately 1.5 m above the ground near the corner of the goat stables. These traps were kept overnight from 6:00 PM to 6:00 AM the following day. Regarding the second method, a mosquito net was hung and covered around the tables where the goats are raised. Anopheline mosquitoes were collected using mouth aspirators (10 mm in diameter × 200 mm in length), and then temporarily stored in paper cups. Mosquito collection by mouth aspirator was from 7:00 PM to midnight^34^.

### Morphological identification, salivary gland dissection, DNA extraction, and amplification of ITS2, *cox1*, and *cox2* genes of mosquitoes

The collected anopheline mosquitoes were identified by groups or species under a stereomicroscope based on the pictorial identification key^14^. For some groups that have a species complex, the mosquitoes were identified up to the group level. The gonotrophic status of the mosquitoes, including unfed, blood-fed, half-gravid, and gravid, was determined. The unfed mosquitoes were observed to have no blood in the abdomen, while those that were fed blood were partially or fully engorged with red blood. The dark red colour of blood covering 3–4 segments and the ovaries/eggs covering the rest of the mosquito abdomen were assigned as half-gravid, while the gravid mosquitoes were blood-free and the ovaries/eggs covered almost all of the abdomen^35^. Anopheline mosquitoes were dissected using a 26G and ½ inch-long sterile needle to separate salivary glands (head and thorax parts) from the midgut (body part). Additionally, 0.1% mercurochrome dye was used to stain sporozoites in salivary glands, after which samples were examined under a microscope at 1,000 times magnification.

Previous studies have revealed that infection can be more easily detected when mosquito samples are pooled^36,37^. Therefore, the anopheline mosquitoes in this study were grouped into pools according to species/group, sampling site, and gonotrophic status. Based on the sample availability of each captured species, a pool contained one to three mosquitoes of the same species/group, sampling site and gonotrophic status. Genomic DNA was extracted from the head and thorax parts of the mosquito that had salivary glands. Non-anopheline mosquitoes were pooled according to genus without dissection. DNA extraction was carried out using NucleoSpin Tissue kit (Macherey–Nagel, Germany) following the manufacturer’s protocol with minor modifications. In the last step, genomic DNA was eluted twice with an elution buffer. The first elution solution was used for the screening of *P. caprae,* while the second was employed for the confirmation of mosquito species. The mosquito species was subsequently identified by a molecular method targeting *cox1*, *cox2* genes, and the ITS2 region. Primers targeting the ITS2 region were described in a previous publication^38^, while primers targeting the *cox1* and *cox2* genes were from a related study^24^. The primers and thermocycling conditions for the identification of mosquito species are described in detail in Table S1.

### Goat blood collection, microscopic examination, and DNA extraction

A total of 401 blood samples were collected from the jugular veins of goats in three provinces of Thailand, including Nan, Kanchanaburi, and Phetchaburi, between 2020 and 2021. Due to the owner’s opposition, goat blood samples in Ratchaburi were not collected concurrently with the time the mosquitoes were captured (June 2021). Therefore, 22 goat blood samples collected from the same goat farm in June 2018 were used for parasite screening. The goat was restrained, then 8 mL of blood was withdrawn, kept in BD Vacutainer® containing 1.5 mL of anticoagulant acid citrate dextrose solution (BD Franklin Lakes, NJ, USA), and brought to the laboratory at the Faculty of Veterinary Science, Chulalongkorn University. Genomic DNA was extracted from 1.5 mL of whole blood using the NucleoSpin® Blood extraction kit (Macherry-Nagel, Germany) following the manufacturer’s protocol.

### PCR detection of *Plasmodium*’s *cytb, 18S rRNA,* and *cox1* genes in mosquito and goat blood

Mosquitoes and goat blood samples were screened for the presence of *Plasmodium* spp. using three sets of primers targeting *cytb*, *cox1* and *18S rRNA* genes. The primers used for the detection of *P. caprae* in goat blood and mosquito samples were described in previous studies^4,24,39,40^. Conventional PCR was applied for confirmation of mosquito species, but nested PCR was required for *P. caprae* detection because parasitemia in the goat blood was extremely low, according to previous results^10^. Each PCR reaction was carried out in a volume of 12.5 µL consisting of 6.25 μL of 2 × buffer KOD FX Neo, 2.5 μL of dNTPs (0.4 mM each), 0.375 μL of each primer (10 pmol/μL), 0.25 μL of KOD FX Neo DNA polymerase (Toyobo, Japan), one μL of the extracted DNA template and 1.75 μL of sterile distilled water. The primers and thermocycling conditions used for *Plasmodium* detection are described in detail in Table S2. *P. caprae* from a previous study was used as a positive control^25^ and sterile distilled water as a no template negative control. All PCR amplifications were conducted in an Axygen® MaxyGene Thermal Cycler (Life science, USA). Gel electrophoresis was set at 100 V, 400 mA, and run for 40 min on 1.5% agarose gel stained with ethidium bromide. The result was evaluated under a UV transilluminator. Regarding the detection of *P. caprae*, the PCR products of positive mosquitoes and blood samples were increased to 50 µL for gel purification and sequencing. Gel purification was performed using NucleoSpin® Gel and PCR clean-up (Macherey–Nagel, Düren, Germany) according to the manufacturer's protocol. The purified samples were bidirectionally sequenced employing the primers used in the second amplification.

### DNA sequences and statistical analyses

The chromatograms of all target genes were carefully checked and then the sequences were manually trimmed and edited using BioEdit v.7.2.5^41^. Low-quality and ambiguous chromatograms were excluded from further analysis. The ClustalW implemented in BioEdit was used to align all obtained sequences then the parasite was identified based on a BLASTN search against the NCBI GenBank database. All consensus sequences were combined with reference sequences retrieved from GenBank. MrBayes v3.2.7 was utilised to construct phylogenetic trees using the Bayesian Inference (BI) and Markov Chain Monte Carlo methods^42^. BI phylogenetic analysis was run for 10,000,000 generations using two independent runs of four chains each. Tracer v1.751 was used to evaluate the mixing and convergence of runs and effective sample sizes (EES > 200)^43^. Furthermore, IQ-TREE was also used for phylogenetic tree reconstruction based on Maximum Likelihood (ML), as previously described^44^. The final phylogenetic trees were visualised and decorated in FigTree v1.4.4 (available online at http://tree.bio.ed.ac.uk/software/figtree/).

The minimum infection rate (MIR) was determined for each mosquito species, in which *Plasmodium* DNA was found to assess the infection rate of positive mosquitoes. It was assumed that a mosquito pool had at least one infected mosquito if *Plasmodium* DNA was found. As a result, MIR was calculated using the formula mentioned: (number of positive pools/total number of mosquitoes studied) × 100^45^. The MIR was estimated using the Wilson confidence interval method for binomial proportions with a 95% confidence interval (CI).

### Ethical statement and biosafety

This study has been approved by the Institutional Animal Care and Use Committee of the Faculty of Veterinary Science, Chulalongkorn University (IACUC No. 2031083). All experiments were carried out according to the Institutional Biosafety Committee (IBC No. 2031037) and university policies and regulations. Goat blood and mosquito samples were collected with the farm owners’ consent. This study was carried out in accordance with ARRIVE guidelines (https://arriveguidelines.org).

## Supplementary Information

Supplementary Tables.

## Data Availability

The nucleotide sequences obtained in this study were deposited in the GenBank™ database (https://www.ncbi.nlm.nih.gov/nuccore) under the following accession numbers: OP271761–OP271765 (*P. caprae*’s *18S rRNA*), OP271766–OP271770 (*P. caprae*’s *cytb*), and OP271771–OP271775 (*P. caprae*’s *cox1*). Nucleotide sequences under accession numbers OP271761–OP271763, OP271766–OP271768, and OP271771–OP271773 were derived from *P. caprae* positive mosquitoes, while the remaining nucleotide sequences originated from goats.
