# Supplementary material for: Myzomyia and Pyretophorus series of Anopheles mosquitoes acting as probable vectors of the goat malaria parasite Plasmodium caprae in Thailand
Source: Sci Rep. 2023 Jan 4;13:145. doi: 10.1038/s41598-022-26833-4 (PMC9812981; doi:10.1038/s41598-022-26833-4)
Supplement: Supplementary file 1 — Supplementary Tables. [file 41598_2022_26833_MOESM1_ESM.pdf]

Table S1. Primer sequences and PCR conditions for mosquito species identification

**Mosquito*<br>(Target gene)**	**Primer name**	**Primer sequence (5'–3')**	**Expected size (bp)**	**PCR condition**
Anopheles spp.<br>(*cox1*)	AnplCox1-F<br>AnplCox1-R	GGATCCCTTCAGCCATTTAATCGCG<br>TCGAGCTTAAATTCATTGCACTAATCTGCC	1,584	94ºC: 5 min<br>**40 cycles: 98ºC: 10 sec, 56ºC: 30 sec; 68ºC: 1 min 40 sec**<br>68ºC: 5 min
Anopheles spp.<br>(*cox2*)	AnplCox2-F<br>AnplCox2-R	GGATCCAGATTAGTGCAATGAATTTAAGC<br>CTGCAGGATTTAAGAGATCATTACTTGC	792	94ºC: 5 min<br>**40 cycles: 98ºC: 10 sec, 58ºC: 30 sec; 68ºC: 50 sec**<br>68ºC: 5 min
Anopheles spp.<br>(ITS2)	ITS2A<br>ITS2B	TGTGAACTGCAGGACACAT<br>TATGCTTAAATTCAGGGGGT	450 – 1,500	94ºC: 5 min<br>**40 cycles: 98ºC: 10 sec, 54ºC: 30 sec; 68ºC: 1 min 30 sec**<br>68ºC: 5 min

Note: *Abbreviation: *cytb*, *cytochrome b*; *cox1*, *cytochrome c oxidase subunit 1*; *cox2*, *cytochrome c oxidase subunit 2*; ITS2, Internal Transcribed Spacer 2; min, minute(s); sec, seconds. Letters in bold indicate amplification steps, including denaturation, annealing, and extension in 40 cycles.

Table S2. Primer sequences and PCR conditions for *Plasmodium* detection in goat blood and mosquito

**Parasite (Target gene)**	**Primer name**	**Primer sequence (5'–3')**	**Expect size (bp)**	**PCR condition**
P. caprae (*cox1*)	Cox1_PbubF (outer)	GTACATTTACTTTTGGTGGTAC	616	94°C: 5 min
				**40 cycles: 98°C: 10 sec, 50°C: 30 sec; 68°C: 40 sec**
	Cox1_PbubR (outer)	CCATCCACTCCATAATTCTC		68°C: 5 min
	PbuCox1-F3-2 (inner)	ATTATGTAATTGCACATTTCCATTTTG	283	94°C: 5 min
				**40 cycles: 98°C: 10 sec, 60°C: 30 sec; 68°C: 20 sec**
	PbuCox1-4B3 (inner)	CCAAATAAAGTCATTGTWGAACC		68°C: 5 min
P. caprae (*cytb*)	DW2 (outer)	TAATGCCTAGACGTATTCCTGATTAT CCAG	1,138	94°C: 5 min
				**40 cycles: 98°C: 10 sec, 62°C: 3 min;** 68°C: 5 min
	DW4 (outer)	TGTTTGCTTGGGAGCTGTAATCATAA TGTG		
	NCYBINF (inner)	TAAGAGAATTATGGAGTGGATGGTG	822	94°C: 5 min
				**40 cycles: 98°C: 10 sec, 62°C: 3 min;** 68°C: 5 min
	NCYBINR (inner)	CTTGTGGTAATTGACATCCAATCC		
P. caprae (*18S rRNA*)	rPLU6 (outer)	TTAAAATTGTTGCAGTTAAAACG	1,200	94°C: 5 min

rPLU5 (outer)	CCGTTGTTGCCTTAAACTTC		**40 cycles: 98°C: 10 sec, 55°C: 30 sec; 68°C: 1 min 30 sec**<br>68°C: 5 min
PlaSSUF1 (inner)	CTTAGTTACGATTAATAGGAGTAG	420	94°C: 5 min<br>**40 cycles: 98°C: 10 sec, 49°C: 30 sec; 68°C: 30 sec**<br>68°C: 5 min
PlaSSUR1 (inner)	TCCTACTCTTGTCTTAAACTAG		

Note: *Abbreviation: *cox1*, *cytochrome c oxidase subunit 1*; *cytb*, *cytochrome b*; *18S rRNA*, *18S small subunit ribosomal RNA*. Letters in bold indicate amplification steps, including denaturation, annealing, and extension in 40 cycles.
